# Peptide Vaccines in Melanoma: Chemical Approaches towards Improved Immunotherapeutic Efficacy

**DOI:** 10.3390/pharmaceutics15020452

**Published:** 2023-01-30

**Authors:** Beáta Biri-Kovács, Zoltán Bánóczi, Anitha Tummalapally, Ildikó Szabó

**Affiliations:** 1 ELKH-ELTE Research Group of Peptide Chemistry, 1117 Budapest, Hungary; 2 Institute of Chemistry, Eötvös Loránd University, 1117 Budapest, Hungary; 3 MTA-TTK Lendület “Momentum” Peptide-Based Vaccines Research Group, Institute of Materials and Environmental Chemistry, Research Centre for Natural Sciences, 1117 Budapest, Hungary

**Keywords:** melanoma, immunotherapy, peptide antigens, adjuvants, nanoparticles, peptide-based vaccines

## Abstract

Cancer of the skin is by far the most common of all cancers. Although the incidence of melanoma is relatively low among skin cancers, it can account for a high number of skin cancer deaths. Since the start of deeper insight into the mechanisms of melanoma tumorigenesis and their strong interaction with the immune system, the development of new therapeutical strategies has been continuously rising. The high number of melanoma cell mutations provides a diverse set of antigens that the immune system can recognize and use to distinguish tumor cells from normal cells. Peptide-based synthetic anti-tumor vaccines are based on tumor antigens that elicit an immune response due to antigen-presenting cells (APCs). Although targeting APCs with peptide antigens is the most important assumption for vaccine development, peptide antigens alone are poorly immunogenic. The immunogenicity of peptide antigens can be improved not only by synthetic modifications but also by the assistance of adjuvants and/or delivery systems. The current review summarizes the different chemical approaches for the development of effective peptide-based vaccines for the immunotherapeutic treatment of advanced melanoma.

## 1. Introduction

Melanoma is developed by the malignant transformation of melanocytes, which are melanin-producing, neural crest-derived cells. It is formed either by the dysfunction of dysplastic nevi or a single melanocyte [1]. Melanocytes are located with the keratinocytes in the basal layer of the epidermis, and they form a very stable population that proliferates extremely rarely under normal circumstances. Not only does the outer layer (epidermis) of the skin contain melanocytes, but also the inner layer (dermis), which involves hair roots, blood and lymph vessels, and nerves; however, they are a biologically different population compared to the ones located in the epidermis. Based on this evidence, cutaneous melanoma is a heterogeneous tumor that involves a wide population of melanocytes with different origins and differentiation stages (from undifferentiated, cancer stem-like cells with self-renewal capacity and high proliferation and differentiation ability to functional melanocytes) [2].

Despite being relatively rare among skin cancers (it accounts for ~21% of all skin cancer incidences), malignant melanoma is very aggressive and was responsible for nearly as many deaths as all other non-melanoma skin cancer types in 2020 [3]. Although early recognized melanoma can be efficiently eliminated by surgery most of the time, the 5-year survival rate for localized melanoma is 99%, while regional occurrence (where lymph nodes are also affected) has a survival rate of only 68%. Moreover, based on its location, it has a high potential to spread rapidly through other body sites via the lymphatic system and the bloodstream. The 5-year survival rate of metastasized, distant melanoma is only 30% (according to the American Cancer Society [4]). 

Significant developments have been made in the treatment of melanoma during the last decade. With the improvement of immunotherapy and targeted molecular therapies, the life expectancy of distant and metastatic melanoma has considerably risen [5]. Despite the recent advances, further progresses and new combinations of existing therapies, as well as new immunotherapeutic strategies, are needed. The current review summarizes the recent advancements in melanoma-focused immunotherapeutic treatments, with a special focus on peptide-based vaccines.

## 2. The Role of the Immune System in Cancers/Melanoma

In 1909, Paul Ehrlich introduced the concept of immune surveillance, which plays a pivotal role in the prevention and repression of tumor progression [6,7]. The relevance of the concept was established by Burnet and Thomas in the late 1950s [7,8]. According to the hypothesis, the immune system can perform the elimination of tumor cells through the recognition of tumor-specific antigens on cancer cells [9]. 

Cancer cells are derived from normal cells that undergo changes leading to uncontrolled proliferation. However, the immune system is not able to distinguish between them, hence their recognition and elimination are minimal [10,11]. Tumor cells that activate the immune system can be easily filtered out; however, tumor cells eventually start to produce specific molecules that are not recognized by the immune system [12]. This process is called immunoediting and has a pivotal role in tumor development due to its successful escape from the defense mechanisms of the immune system.

**Immunoediting** can be divided into three phases: elimination (recognition of the tumor by the adaptive immune system), equilibrium (state of cancer persistence, in which a balance is formed between the eliminated and the newly developed tumor cells), and escape (phase of cancer progression that results in poorly immunogenic tumors and during which metastases are developed).

In the **elimination** phase (which agrees with the concept of immunosurveillance), tumor cells can be successfully recognized and eradicated by innate and adaptive immune system components. Transformed cells initiate stromal remodeling that results in local tissue disruption, which is recognized as a danger signal by natural killer (NK) cells, T cells, and macrophages. These adaptive immune system cells produce cytokines, such as interferon-γ (IFN-γ) and other interleukins (ILs), to trigger extrinsic tumor suppressive mechanisms which result in the death of tumor cells [13]. This tumor cell killing effect is performed in different ways: (i) the activation of antiproliferative, proapoptotic, and angiostatic processes by IFN-γ, (ii) the production of reactive oxygen species (ROS) and reactive nitrogen intermediates by macrophages, and (iii) via TRAIL (TNF (Tumor necrosis factor) Related Apoptosis Inducing Ligand) or perforin-dependent mechanisms by NK cells [14]. In parallel, antigen presenting cells (APCs), such as dendritic cells (DCs), are recruited at the tumor site and activated by tumor antigens generated during the elimination procedure. DCs take up tumor antigens and migrate to lymph nodes to activate the IL-2-producing CD4^+^ (Th1) and tumor-specific CD8^+^ (Th2) T cells, which migrate to the tumor site and eradicate the viable antigen-positive tumor cells. IL-2 is essential for maintaining the activated state of CD8^+^ T lymphocytes. CD8^+^ T cells can directly recognize and eradicate tumor cells by the above, detailed IFN-γ dependent mechanism or by induction of macrophages [13].

If the elimination of tumor cells is not completely achieved (e.g., some tumor cell variants are resistant to elimination), the **equilibrium** phase may develop. In the prolonged equilibrium phase, which may take years, the elimination of tumor cells and the production of resistant variants occur. The tumor growth is controlled by the adaptive immune system, although it is not able to conclusively eliminate cancer cells. Adaptive T cells have a key role in the activation and maintenance of the dormant-like equilibrium phase [15]. Moreover, tumor-infiltrating lymphocytes might help to reduce the tumor progression in many cases (e.g., ovarian [16], colon cancer [17], etc.). On the one hand, tumor cells can maintain memory T cells and persistent antigens at a relatively low level, which mediates CD4^+^ and CD8^+^ related responses [18]. This evidence can be utilized in adoptive cell-transfer (ACT) therapy in advanced-stage cancer, especially in melanoma [19]. On the other hand, during this process, tumor cells progressively lose MHC I molecules, which significantly increases the importance of cytotoxic CD8^+^ T lymphocytes to prevent cancer progression [13,20]. In the equilibrium phase, tumor cells become more immunogenic, and tumor antigens play a role in deciding whether to promote or inhibit tumor progression [13]. Unfortunately, in the tumor microenvironment, not only the elements of the immune system induce changes, but also the tumor itself. Tumor cells produce and release immune system blocking molecules; therefore, the immune response is selectively suppressed around the tumor microenvironment [21,22]. In addition, inflammation due to the tumor development can also release many immunosuppressive cytokines locally in the tumor, which suppress the immune system, thus, promoting tumorigenesis [12]. Taken together, the tumor might often overcome the immune defense and enter the next phase (the escape phase). 

A heterogenic tumor cell population can be formed with a different set of genetic and molecular alterations, resulting in the loss of immunogenicity of tumor cells and a significantly altered microenvironment. The changes occurring due to dysregulated cytokines, increased proteolysis, hypoxia, phagocytosis of apoptotic tumor cells, and FC receptor crosslinks together lead to the development of the immunosuppressive state, which is a precursor to the **escape phase**. Typically, in this phase, the tumor progresses, metastases develop, and the clinical symptoms reappear [13,23,24,25,26]. Briefly, a wide set of processes make the tumor invisible to the immune system: (i) shedding of antigens and losing MHC on tumor cells; (ii) downregulation of adaptive and innate immune responses by secretion of cytokines (TGF-β, IL-10); (iii) induction of CTLA-4 (cytotoxic T lymphocyte associated antigen-4) or regulatory T cells [27,28]; (iv) production of myeloid suppressor cells (MSCs) with immune suppressive function; (v) expression of aberrant antigens on tumor cells (e.g., HLA-G); (vi) resistance to apoptosis and vascularization by VEGF. Together, these events eventually result tumor growth, invasion, and the development of metastases [13].

## 3. Immunotherapeutic Approaches for the Treatment of Melanoma

Due to its protective feature, the skin also functions as an immunological barrier to protect the body from environmental risk factors, such as UV irradiation, chemicals, and pathogens [9]. Since gaining a deeper understanding of the mechanisms of melanoma tumorigenesis and their strong interaction with the immune system, the development of new therapeutic strategies has been continuously rising. It is also well shown by the number of approved therapeutic approaches for melanoma treatment. In general, FDA-approved therapeutic strategies can be divided into two main categories: immunotherapy and targeted therapy. The list of approved drugs confirms the relevance of the application of immunotherapy for melanoma, namely that the number of these types of drugs is growing year by year. The FDA-approved approaches involve drugs or vaccines for immunotherapy, targeted therapy, and combination therapy (a combination of immune and targeted therapy). Immunotherapeutic drugs can be differentiated as (i) immune checkpoint inhibitors (e.g., programmed death 1 (PD-1) and its ligand (PDL-1), cytotoxic T lymphocyte-associated 4 molecule (CTLA-4); (ii) non-specific immunomodulation by cytokines (e.g., IL-2, IFN α-2b); (iii) application of tumor vaccines or oncolytic viruses, and (iv) usage of adoptive T cell transfer [29].

This review focuses on peptide-based immunotherapeutic approaches, especially tumor vaccines; therefore, other strategies are only briefly discussed below. 

The high number of melanoma cell mutations induced by UV irradiation provides a diverse set of antigens that the immune system can recognize and use to distinguish tumor cells from their normal counterparts. The antigen recognition-induced T cell response is strongly dependent on the balance between co-stimulatory and inhibitory signals. These signals are named immune checkpoints [30,31]. Mapping the signaling pathway of immune checkpoint receptors opens a new door for the treatment of melanoma. Two main immune checkpoint molecules, CTLA-4 and PD-1, have a central role in the regulation of immune responses. Their expression is often dysregulated on tumor cells or immune cells, preventing the eradication of tumor cells by effector T cells. Therefore, the effect of their blockage on tumor progression has been intensively studied. Since the introduction of the concept of immune checkpoint blockade for the treatment of cancer by Allison et al. [32], three immune checkpoint inhibitors have been approved by the FDA for melanoma treatment: two against CTLA-4 (ipilimumab (Yervoy, approved in 2011) and Tremelimumab (Imjudo, approved in 2022) [33]), and two against PD-1 (pembrolizumab (Keytruda, approved in 2017) and nivolumab (Opdivo approved in 2014), respectively), and one against PD-L1 (Atezolizumab (Tecentriq, approved in 2016) [34]. All of them are humanized monoclonal antibodies that block the interaction between the receptors and their ligands. The physiological role of **CTLA-4** might be to prevent the development of autoimmunity, and this function also probably plays an important role in the neutralization of antitumor immune responses. CTLA-4 has a negative effect on T cell proliferation, on IL-2 production, and the expression of its receptor, and promotes cell cycle arrest at G1 as well [35]. According to the experimental results obtained by in vitro and in vivo studies, as well as clinical trials, blockage of CTLA-4 is an effective way for melanoma treatment. **PD-1** has a central role in immunopathology and tumor immune surveillance due to its effector T cell inhibition [36]. Physiologically, PD-1 (like CTLA-4) is expressed on T cells and promotes T cell anergy, apoptosis, and exhaustion [37]. Nowadays, there is intensive ongoing research to find other checkpoint molecules that can be potential targets for immunotherapy. The next generation of immune checkpoint inhibitors is systematically summarized in the literature [34,38]; therefore, this review is not detailing them.

Non-specific immunomodulation can be obtained by the administration of **cytokines**. As we mentioned previously, cytokines are essential for maintaining the activated state of T lymphocytes, which directly recognize and eradicate tumor cells [13,14]. Some of the cytokines have their own antitumor effect; they can directly inhibit the growth of tumor cells. Nevertheless, the secretion of several cytokines is suppressed in tumors; therefore, the administration of cytokines seems plausible to neutralize the tumor induced immunosuppressed state. However, intensive research is underway to determine which cytokines are the most favorable for immunotherapy. Currently, only two have been approved for melanoma treatment by the FDA; high-dose IL-2 (HD-IL-2) for metastatic melanoma treatment and INF-α as adjuvant therapy for stage II and stage III melanoma. Regrettably, the application of these cytokines is controversial due to their short half-lives and the toxicity caused by high and frequent dosages. Many attempts have been made to replace them (e.g., fusion, recombinant, or chemical modification) and develop a safe and effective cytokine derivative with improved pharmacokinetic properties [39]. Current results obtained with cytokines are summarized in several recent reviews [39,40].

Since the discovery of the close connection between tumor infiltrating lymphocytes (TILs) and IL-2, it has been well accepted that tumor-reactive T cells can significantly mediate tumor regression [41]. TILs, as a heterogenous population, consist of mainly T cells and NK cells, which are capable of recognizing tumor-associated antigens (TAAs). Therefore, the presence of TILs is closely associated with a favorable prognosis for cancer. Briefly, in **adoptive T cell transfer** (ACT) strategies, T cells are manipulated ex vivo and reinfused into the patient. Natural tumor-specific T cells (isolated from tumor cells) and genetically modified T cells (which can specifically recognize tumor cells) can be utilized for ACT. Unfortunately, TILs cannot be isolated from all patients; therefore, the application of expanded tumor-reactive T cells is needed. On the other hand, not all melanoma patients produce anti-melanoma T cells due to the shedding process of tumor cells (detailed in Session 2). In this way, cloned T cell receptors (TCRs) or chimeric antigen receptors (CARs), isolated from melanoma-reactive T cells, can be used to avoid the failure of immunotherapy. For genetic modification of T cells, several commonly used gene transfer methods can be applied, such as transient mRNA transfection [42], viral vectors [43,44], transposons [45], and homologous recombination after gene editing [46]. Even though ACT is mainly used in late-stage melanoma after the failure of standard therapies, complete regression of the tumor is observed in patients treated with TIL, and recurrence is not observed even many years after the treatment. Independent studies, obtained from the usage of TILs, clearly demonstrate their relevance and potential in the clinical application of ACT. As a result, personalized cancer immunotherapy is a hot topic; it is rapidly developing due to the increasing number of clinical trials to enhance its efficacy and extend its application to other cancers with frequent mutations [47].

## 4. Types of Antigens: Overexpressed Antigens, Cancer Testis Antigens, Mutated Oncogenes and Patient-Specific Mutated Neoantigens

**Figure 1:**
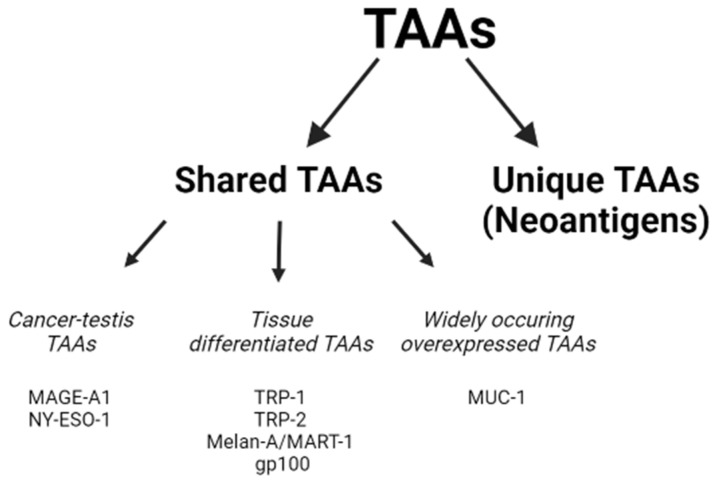
Classification of tumor-associated antigens (TAAs).

The high number of mutations during tumorigenesis results in a myriad of potential antigens for each individual cancer. Classically, tumor antigens can be divided into three groups: (i) tumor-associated antigens (TAAs), (ii) cancer-specific antigens (also called neoantigens), and (iii) cancer testis antigens (CTAs). Due to the high number of already described and constantly newly characterized TAAs, the classical grouping is restructured into two main classes, namely the shared and the unique **TAAs** (Figure 1). The continuously updated list of these antigens can be found at http://www.cancerimmunity.org/peptide/ (accessed on 30 November 2022) [48].

**Shared TAAs** consist of three groups: cancer testis, tissue differentiation, and widely occurring overexpressed antigens. *Cancer testis antigens* are derived from the reactivation of physiologically silent genes, and they can be activated in different tumor histotypes. MAGE-A1 [49,50] and NY-ESO-1 [51] are the most prominent members of CTAs. They are mostly expressed in the placenta, testis, and some malignant cancers. Placenta and testis cells do not express MHC class I molecules to present antigens on their cell surfaces. Therefore, T cells specific to cancer testis antigens attack only cancer cells and hence become ideal targets for T cell-based immunotherapy. Since 1991, when melanoma-associated antigen 1 (MAGE-A1) was first described [52,53], several cancer testis antigens have been discovered and intensively studied as targets for immunotherapy. MAGE-A1 is a highly expressed antigen, as it can be identified in about 50% of melanomas and around 10 to 50% of other cancers [19,52]. 

*Tissue differentiation antigens* are normal, non-mutated proteins that are shared between the tumor and the normal tissues from which the tumor arose. In the case of melanoma, these antigens are present on normal melanocytes and melanoma cells as well, and are recognized by T cells when they are processed and presented on the cell surface by MHC molecules. These antigens are termed differentiation antigens and include tyrosinase, tyrosinase-related proteins (TRP-1 and TRP-2) [54], melanocyte antigen (Melan-A/MART-1) [55], and glycoprotein 100 (gp100) [56,57,58]. Since these antigens are not tumor-specific, targeting them can cause severe side effects and toxicities [59]. 

*Widely occurring overexpressed antigens* are typically overexpressed by tumor cells; however, their low expression level was observed in normal cells. They reach the threshold for T cell recognition to break the immunological tolerance and trigger an anticancer response. Mucin 1 (MUC1) is representative of this antigen category. MUC1 is a unique antigen, as it is not only overexpressed but its glycosylation status is also altered in tumor cells. Hence, it is an attractive target for cancer immunotherapy [48]. 

Random somatic point mutations (e.g., single nucleotide variation; deletions, fusions, or alternative splicing; alterations in the post-transcriptional or post-translational steps), caused by carcinogens, result in **unique TAAs**, hence neoantigens. These neoepitopes are presented either by the tumor cells or by APCs and are recognized by T cells [60,61]. Neoantigen-reactive T cells kill tumor cells in an antigen-specific manner. As they are highly tumor-specific, by targeting these antigens, the likelihood of toxic effects is very limited. Therefore, they are ideal targets for cancer immunotherapy [48,62,63]. Neoantigens can be classified into two categories: shared and personalized neoantigens. The shared ones are highly immunogenic antigens that are identical in several groups of cancer patients and are not present in the genomes of normal patients. Their identity makes them suitable as antitumor vaccines against cancer in patients with the same mutation. Personalized neoantigens are unique, varying from patient to patient. Thus, these neoantigens can only be used as vaccines in specific, personalized therapies [12]. It is worth noting that not all mutations result in antigens with highly immunogenic features. Most of them do not affect the tumor development or progression, thus they have no relevance as vaccine targets [48]. Neoantigen vaccines have been tested for melanoma in three clinical trials; two of them used synthetic peptides to minimize formulations [64,65], and the third one used synthetic RNA [66]. Though personalized immunotherapy has huge relevance in melanoma treatment, the application of neoantigens as cancer vaccines is often time-consuming and expensive [64,67]. 

## 5. Peptide Vaccines: Benefits, Drawbacks, Types and Composition

**Figure 2:**
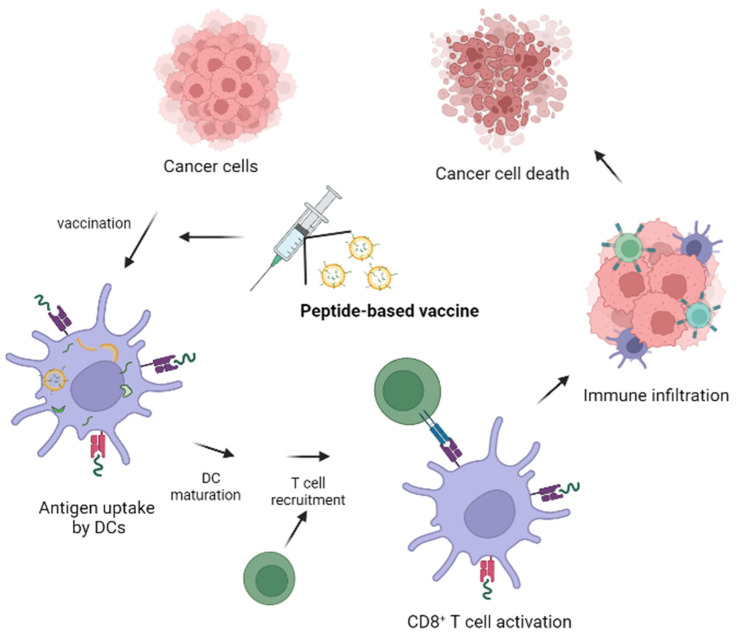
Mechanism of action of peptide-based vaccines.

Cancer vaccines have emerged and developed rapidly in the last few decades, and we can currently distinguish several types: peptide vaccines, dendritic cell vaccines, mRNA vaccines, DNA vaccines, and viral vaccines as well. All of them are based on tumor antigens that elicit immune responses due to APCs. APCs, such as DCs, are capable of inducing cell-mediated immunity by capturing proteins expressed by viruses or tumor cells, internalizing them and fragmenting them into short peptides. These peptide fragment sequences are the potential antigens that should be used for the development of cancer vaccines. Therefore, targeting DCs is the most important possibility for vaccine development, which should be achieved by several kinds of chemical alterations of possible antigens, such as modification of their structure, using conjugation strategies, and their encapsulation into nanoparticles [68,69]. The mechanism of action of peptide-based anti-tumor vaccines is summarized in Figure 2.

Peptide-based vaccines are produced almost exclusively by synthetic methods and can be fully and accurately characterized as chemical entities. Due to synthetic approaches, the production of peptide vaccines is simple, easy, and reproducible. Solid-phase peptide synthesis (SPPS) is a fast and cost-effective way to obtain peptides on a large scale. Moreover, the automated flow peptide synthetic method (which is a newly developed subtype of SPPS) can produce neoantigens of higher quality within a maximum of 35 min compared to the classical SPPS methods, which take hours or days [70]. Chemical characterization of peptides, as well as peptide vaccine constructs, can be performed by using well-established analytical methods, such as high-pressure liquid chromatography (HPLC) and mass spectrometry (MS). The peptide composition and content of the vaccines can be exactly determined by amino acid analysis [71,72].

The introduction of non-natural amino acids and peptidomimetics into peptide-based vaccines allows the design of more drug-like compounds, which opens a new way for vaccine delivery and rational drug design in cancer vaccination [73]. Unlike expression techniques, chemical synthesis results in pure peptide vaccines that are free of biological contaminants and elicit a specific immune response without triggering an allergic or autoimmune reaction [74]. Due to the chemical structure of amino acids, lipids such as fatty acids, carbohydrate groups, and phosphate groups can be incorporated in an easy and controlled way to improve their immunogenicity, stability, and solubility. By encapsulation into nanoparticles, peptide vaccines can become water-soluble and stable under standard storage conditions; they can be often safely stored as a powder after lyophilization. On the other hand, the use of peptide vaccines totally excludes genetic integration or recombination, which is a relevant problem in DNA vaccines [74].

It is worth noting that despite the synthetic advantages, peptide antigens are poorly immunogenic themselves and require the assistance of adjuvants and/or delivery systems. Their peptide nature is a double-edged sword: their susceptibility to enzymatic degradation is both, a disadvantage because it makes them difficult to use without any modification, and an advantage because they are completely biodegradable. Thus, during their degradation process, no harmful metabolites are released and accumulate in the body after the treatment; however, their degradation may produce unrelated immune responses. Additionally, the population coverage of peptide antigens varies due to the large number of mutations; hence, they are often not uniformly recognized by the whole human population [12,74]. 

To overcome the low immunogenic activity of single epitope vaccines, multiepitope vaccines are manifesting as the next generation of cancer immunotherapy. Multiepitope vaccines might consist of the inclusion of epitopes with adjuvant effects, the incorporation of immunodominant epitopes (that might come from the same protein or different proteins), and the omission of immunologically irrelevant epitopes. On the other hand, multiepitope vaccines may overcome some potential safety concerns and seem to be able to elicit stronger immune responses compared to whole antigen immunization [73,74,75]. The main advantages and drawbacks of peptide-based, synthetic anti-cancer vaccines are summarized in Table 1. 

## 6. How to Improve the Peptide?

To decide which antigens should be used as a vaccine, different bioinformatic algorithms and databases (such as NCBI database [76], Immune epitope database (IEDB [77]) can help predict their antigenicity (Vaxijen v2.0 server [78]), physicochemical features (Expasy [79]), proteasomal cleavage (NetChop 3.1 server [80]), TAP binding predictions (TAPREG [81]) and in vivo behavior (C-ImmSim [82]) as well. As a result of new-generation sequencing (e.g., mass spectrometry) and computational prediction methods, the genetic alteration of tumor cells encoding specific mutant peptides can be easily identified. Because of their specific ability for T cell induction and activation, they can be used as vaccines for personalized immunotherapy [12].

As we mentioned above, peptide antigens are poorly immunogenic themselves and require not only synthetic modification to improve their immunogenicity, but also the assistance of adjuvants and/or delivery systems. In this session, we list in detail the possible chemical modifications that can be carried out to enhance the antigenicity of peptide antigens. 

Improving the immunogenicity of an antigen can be very challenging. Antigens, especially neoantigens, often contain only a small alteration compared to native proteins and peptides. Thus, their modification may jeopardize their in vivo efficacy. Moreover, the activation of the immune system by antigens has many steps, from the uptake of antigens by antigen presenting cells to the presentation and recognition of the peptide-MHC I complex by T cells. Because of this complexity, improving one aspect does not necessarily mean enhancing the whole process. 

As short peptides have many drawbacks as T cell stimulator antigens, they are commonly incorporated into nanocarriers. If this happens via physical interaction, it does not need chemical modifications. Even if this approach is easier from a chemical aspect, it has severe restrictions. Because of the high hydrophilicity of antigen peptides, there is a strong limitation to their encapsulation in nanocarriers. For example, nanoencapsulation of two murine melanoma antigenic peptides, gp100(25–33) (KVPRNQDWL) and TRP-2(180–188) (SVYDFFVWL) by biodegradable poly(D,L-lactide-co-glycolide) (PLGA) nanoparticle, showed very different efficacy depending on the hydrophilicity of the two peptides. The encapsulation efficacy of a more hydrophilic gp100(25–33) peptide was very low, and it had faster in vitro release [83].

### 6.1. Enhancing the Binding Affinity

Several excellent examples point out the importance of the binding affinity of antigens to the corresponding MHC receptor type. By chemical modification, the binding affinity of peptide antigens can be easily and efficiently enhanced, as clearly demonstrated by the following publications. Two melanoma associated antigens (Melan-A(27–35): AAGIGILTV and Melan-A(26–35): EAAGIGILTV) were studied to improve their binding to MHC I (HLA-A2) with a single amino acid substitution [84]. The binding of these two antigen peptides is not optimal because they do not have the proper amino acid (Leu or Met) at position 2 (the dominant anchor amino acid residue) [85]. In the case of the nonapeptide, both the Ala-Leu or Ala-Met replacement at position 2 increased binding to the HLA-A2 molecule; however, it dramatically decreased their antigen activity. When Ala at position 1 was exchanged for Leu or Met, the binding was not improved, but the peptide recognition was increased. In the case of the decapeptide, the Leu at position 2 increased both binding and peptide recognition. The best construct was the E**L**AGIGILTV analog (ELA peptide), which had higher immunogenicity than those of the native peptides. This peptide was further improved: its enzymatic stability was increased, and its high immunogenicity was retained [86]. It turned out that modification of both termini is necessary for efficient protection against enzymatic degradation. While the peptide bond modification was deleterious, using modified amino acids such as α-methylated, N-hydroxylated or β-amino acids was efficient to protect against proteolysis while retaining the peptide antigenicity. Later, the central part of this peptide, the TCR-contacting portion, was successfully modified by a nonpeptidic moiety to increase its activity [87,88]. A derivative with an indole acetic acid sidechain resulted in an increased T cell response. In another study, a different side chain was introduced into this unit of ELA mimicking peptide using copper(I)-catalyzed azide-alkyne Huisgen 1,3-dipolar cycloaddition [89]. When the HLA-A2 binding activity and antigenicity of these new derivatives were compared, there was no correlation: the compound with weak binding ability was better recognized by T cells. These results demonstrate that the binding affinity to MHC I proteins does not predict the immunogenic potency [89].

Studies focusing on improving the binding of epitopes to MHC I and thus enhancing their immunogenicity are summarized by Slansky and Nakayama [90]. Although these modifications can be useful to identify new neoantigens [91], sometimes the increased binding to MHC I does not result in enhanced immunogenicity, and many times the modified peptides can be discriminated as different antigens from their wild-type counterparts. Although modification of anchor amino acids is not supposed to change the binding because it is buried from the T receptor binding site, it may behave as an allosteric modifier of T cell receptor binding [92].

### 6.2. Usage of a Covalent Linkage between the Antigen and the Nanocarrier

**Figure 3:**
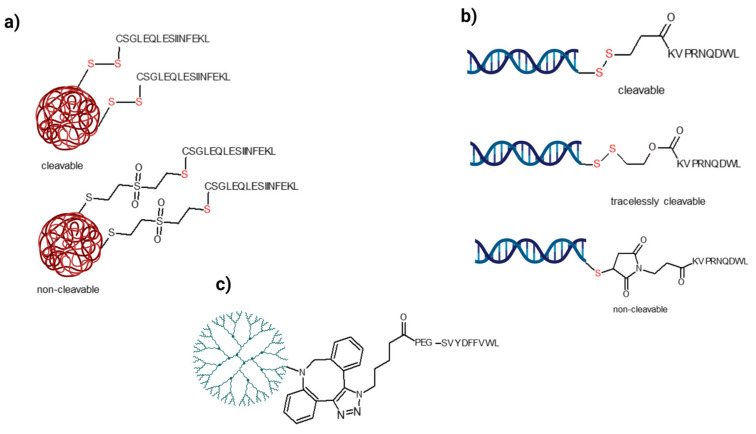
Different approaches for antigen-peptide conjugation. (**a**) Usage of thiol chemistry to form cleavable or non-cleavable covalent linkage, (**b**) usage of thiol chemistry to form cleavable, tracelessly cleavable, or non-cleavable connection, and (**c**) usage of click chemistry for conjugation.

The other way of nanoformulation is chemical modification, and thus conjugation of the antigen peptides to the nanocarrier or one of its components. Although attachment of antigens to nanocarriers gives a good opportunity to increase the amount of peptide, it may be challenging as well. The chemical modification of antigen peptides may influence the processing and chemical structure of the antigen. The efficiency may depend on the type of covalent linkage, which can be cleavable or non-cleavable. The disulfide bond can be cleaved intracellularly by the glutathione system, allowed by the reductive environment inside the cell. When ovalbumin (OVA) peptides as model antigens were conjugated to the nanocarrier via a covalent bond, the construct with a reducible bond was more efficient than the construct with a nonreducible bond (Figure 3a) [93]. For disulfide bond formation, “only” an extra cysteine should be inserted at one terminus of the peptide. Hence, these OVA peptides were extended at their *N*-terminus with a Cys residue. This conjugation strategy was also used in the case of the gp100(25–33) peptide [94]. The Cys was introduced to its *C*-terminus and then it was conjugated to the thiol-containing modified PLGA. In another study, the effect of the linker on the activity was studied. The gp100(25–33) peptide was covalently attached to the spherical nucleic acid via three different linkers [95]. First, the α-amino group of the peptide was acylated with three different thiol-reactive linkers. Then the modified peptide was conjugated with nucleic acid using the thiol-reactive functional group of the linker. Three different types of connections were used: non-cleavable, tracelessly cleavable (after the reduction, the linker is removed from the *N*-terminus of the peptide), and cleavable (the reduced linker remains on the *N*-terminus of the peptide) (Figure 3b). The traceless linker was the most potent (8-fold improvement in T cell proliferation), highlighting the importance of the selected linker in this strategy. Similarly, melanoma-specific antigens (TRP-1, TRP-2, gp100, and MART-1 derived peptides) with Cys at their *C*-terminus were conjugated to antibodies using the sulfhydryl-maleimide reaction [96]. In another example, strain promoted azide-alkyne click chemistry was used to synthesize an antigen-containing nanoconstruct [97]. TRP-2(180–188) was modified with azidovaleric acid via a PEG spacer at its *N*-terminus. In this construct, the antigen could not be released intracellularly because of the non-cleavable covalent bond (Figure 3c). A similar strategy was used to conjugate OVA (257–264), OVA(323–339), and M30 (PSKPSFQEFVDWENVSPELNSTDQPFL) [98]. First, the 𝛽-1,3-glucan particles were modified by epoxy-PEG_4_-N_3_, then the *N*-terminal DBCO-modified (dibenzocyclooctyne) antigen peptides were attached to these linkers.

To simplify these constructs, the antigen peptide can also be modified to get a derivative that can self-assemble into a nanocarrier. Human gp100(25–33) epitope peptide was modified at its *N*-terminus with a hydrophilic PEG_8_ and a hydrophobic photosensitizer, protoporphyrin IX [99]. This amphipathic structure can form nanostructures in the aqueous phase. In another report, the EGFRvIII peptide (LEEKKGNYVVTDH) as a neoantigen from the *N*-terminal of mutant EGFR was conjugated with the PADRE (Pan DR helper T cell epitopes) peptide via its *N*-terminus using amide bond formation. This chimera peptide was then attached to cholesterol using a succinic acid spacer. This structure could form a micelle, which produces a high immune response [100].

### 6.3. Self-Assembling Moieties for Improved Efficacy

Not only hydrophobic moieties may induce self-assembly of the antigen construct, but peptides also tend to form nanostructures. For instance, nanoscale structure formation was observed in the case of the peptide KKFKFEFEF in an aqueous solution coupled to the TRP-2 antigen via a disulfide bridge. For conjugation, a Cys was introduced to the *N*-terminus of the antigen peptide, and the conjugate transformed into a hydrogel when a few drops of salt were added [101]. A similar attempt was made to modify the epitope peptide to get a nanocomplex by Shi and co-workers. They developed a nanocomplex vaccine based on a spontaneous non-covalent interaction between the CpG and the cationic oligoarginine elongated epitope peptide. To establish the efficacy of the nanovaccine, the nanocomplex preparation was carried out without CpG. By itself, the conjugation of octaarginine to the *C*-terminus of the TRP-2 antigen does not result in a self-assembling peptide. However, the octaarginine-elongated epitope peptide with the CpG (as a negatively charged molecule) spontaneously forms nanocomplexes via electrostatic interaction. This vaccine activated DCs and elicited comparable therapeutic antitumor efficiency [102].

### 6.4. Incorporation of Lymph Node Targeting Moieties

Antigen efficacy may be improved by binding to endogenous proteins, thus increasing its half-life and targeting it to lymph nodes. A systematic study identified diacyl glycerol as an ideal binding partner of albumin [103]. Modification of peptides with this highly hydrophobic moiety may result in very low water solubility. To avoid this, a hydrophilic polyethylene glycol block was introduced into the construct. The antigen peptide was extended by an extra Cys at its *N*-terminus and conjugated with an amphiphile diacyl glycerol-PEG tail using maleimide-thiol chemistry. During further optimization of this construct, the diacyl glycerol moiety was replaced by an albumin binding cyclic peptide and E-vitamin (α-tocopherol). These novel analogs had the same efficacy [104]. Three conjugation techniques, thiol-maleimide, strain-promoted alkyne azide cycloaddition (SPAAC), and amide bond formation, were used and their effects on the activity were compared. Except for amide bond formation, the antigen peptide (gp100(20–39): AVGALEGPRNQDWLGVPRQL) was modified on its *N*-terminus (extra Cys or 5-azidopentanoyl group) for the conjugation reaction. Evans Blue (EB) and its derivatives can bind to serum albumin and may be used to design albumin binding antigen constructs. When a melanoma-associated subunit Ag, TRP-2(180–188), was attached to a maleimide-functionalized EB derivative, the peptide was extended with a Cys at its N-terminus. This construct formed self-assembled amphiphilic nanoparticles in an aqueous solution and was efficiently accumulated in lymph nodes via albumin binding, which significantly prohibited tumor growth [105].

### 6.5. Usage of Cell-Penetrating Peptides

There may be several reasons why a peptide antigen may not be efficient enough *in vivo*. One possible explanation could be its poor presentation by MHC I, which is associated with low cellular uptake. Although the internalization of antigens into DCs may be enhanced by nanoparticles, their fate is often degradation in endolysosomes. To avoid this unwanted degradation, the use of carriers with pH-dependent membrane destabilizing activity may solve the problem and promote an immune response. When cell-penetrating peptides (CPP) were conjugated to gp100(20–38) antigen peptide, the immunogenicity was increased in vivo by as much as 25-fold. Similar to albumin binders, the CPP has resulted in increased accumulation in lymph nodes. In addition to the different CPPs, the influence of structure and conjugation mode was also studied. It turned out that neither the connection of the antigen and the CPP (C-N or C-C terminus), nor the type of the covalent linkage (azide/alkyne click chemistry or synthesis as a single continuous sequence) influences the activity of the constructs; all of them had the same behavior [106]. In another related publication, electrostatic self-assembly was achieved using polyanionic poly (propylacrylic acid) and polycationic peptide antigen, showing enhanced immunogenicity in a neoantigen cancer vaccine [107]. To reach the polycationic form, the antigen peptide was extended at its *N*-terminus by decalysine. The cellular uptake may be enhanced by conjugation with a cell-penetrating peptide as well. A cell-penetrating peptide variant of the protein ZEBRA was conjugated with epitope peptides via an amide bond. Using the proper adjuvant, these conjugates showed potent antitumor immunity [108].

These examples show that although immune recognition is very sensitive to the chemical structure, there are possibilities for chemical modification and thus improvement of the epitope peptides. One bottleneck of this strategy is its unique and often unpredictable effect. There is not a generally applicable modification, but there are possibilities that may be tried for better efficacy.

## 7. Adjuvants and Formulation

The main requirement for antigens is their strong potency for TLRs or MHC receptors to promote the activation of CD8^+^ and CD4^+^ T cells to induce an immune response. Peptide antigens correlating with MHC I and CD8^+^ T cells are typically 8–10 amino acids long, while antigens for MHC II receptors and CD4^+^ T cells are usually 13–18 amino acids long [109]. As these small peptide antigens have poor immunogenicity, the use of adjuvants is essential for eliciting a high-frequency immune response. Unfortunately, adjuvants alone cannot increase the immunogenicity of peptide antigens, the use of delivery systems is usually required to achieve the desired response. Therefore, peptide vaccines consist of three main components: epitope peptides (single- or multiepitope), adjuvants, and a delivery system (namely nanoparticles). The main drawback of the adjuvants is their structural variability, which produces a huge challenge for the administration and process comprehension [110].

### 7.1. Adjuvants in Melanoma Vaccines

Since the license for human usage of the first adjuvant, aluminum salts (generally referred to as *Alum*), a continuously growing number of adjuvants have been reported in parallel with the development of vaccines. Various adjuvants have been evaluated in preclinical, as well as clinical trials to overcome the immune tolerance of peptide vaccines. Here, we briefly list the main adjuvant groups and their most prominent representatives, which have been used in preclinical or clinical trials. In general, they can be classified into two main groups: molecular adjuvants and immunostimulating complexes [111].

#### 7.1.1. Alum

The immunization-enhancing effect of Alum was first reported in the early 1930s [112,113]. Since its discovery, Alum has been commonly used as an adjuvant in human licensed vaccines such as influenza, tetanus, diphtheria, pertussis, poliomyelitis, and HPV [114,115,116]. Alum can also be found in clinical trials for testing melanoma peptide vaccines. Hamid et al. performed a trial involving sixty patients (with high-risk resected melanoma), in which three peptide epitopes (TYR(368–376), MART-1(126–135), and gp100(209–217) were combined with IL-2 and Alum or IL-2 and GM-CSF. They found that the IL-2/Alum combination resulted in a more beneficial immune response compared to the IL-2/GM-CSF [117]. However, adverse and toxic side effects have been reported with the use of aluminum adjuvants [113,118,119].

#### 7.1.2. Emulsions 

Emulsions are typically a mixture of two immiscible liquids, resulting in water-in-oil or oil-in-water emulsions [120]. The first approved emulsion for human use was MF59, in an influenza vaccine [111]. Since the discovery of MF59, emulsion-like adjuvants have also been continuously developed. QS-21 is a purified saponin extract from *Quillaja saponaria*; it has limited toxicity and promotes both CD4^+^ and CD8^+^ immune responses [120]. The 3-O-desacyl-4′-monophosphoryl lipid A (MPLA) is a lipopolysaccharide derivative that has low toxicity and can activate both B cell and T cell responses due to its TLR 4 agonist property [121]. The combination of QS-21 and MPLA results in a new oil-in-water emulsion, AS02. It was tested in a clinical trial for the Melan-A/MART-1(26–35) peptide vaccine in melanoma patients. The immunization resulted in antigen-specific CD8^+^ activation and the induction of memory T cells, respectively [122]. Due to the essential and required role of TLRs in adaptive immune responses, several kinds of molecules have been recognized as TLR ligands (e.g., LPS, poly I:C, and CpG-DNA) [123]. Two prominent representatives of TLRs have a key role in melanoma immunotherapy: TLR 9 and TLR 7. The Uumethylated cytosine–phosphate–guanine (CpG-DNA) oligonucleotide family involve a series of CpG-DNA variants with a bacterial origin, which are TLR 9 agonists. They can activate both DCs and B cells to produce CD4^+^ cytokines [124]. Imiquimod, resiquimod, and loxoribine are special agonists for TLR 7 [125,126].

#### 7.1.3. Freund’s Adjuvants

Freund’s adjuvants exist in completed (CFA or FCA) or incomplete (IFA or FIA) forms; they are popular delivery methods for various peptides [127,128]. IFA is identical to CFA but without the heat-killed *Mycobacteria* component. Both are often used to increase humoral and cellular immunity in animals and humans [129,130,131]. Montanide is a water-in-oil adjuvant family. One of its members, ISA-51, is so-called incomplete Freund’s adjuvant (IFA) is commonly used in cancer vaccines [132]. They facilitate the depot effect at the site of vaccination and provide continuous antigen exposure in the stabilized oil emulsion [133]. Chu et al. developed an amphiphilic liposome-based nanovaccine with lymph node targeting characteristics that is composed of neoantigens (Tyrp1: TAPDNLGYM, M20: FLHWYTGEAMDEMEFTEAE, M27: LCPGNKYEM) and Montanide^TM^ ISA-51. The construct was tested in B16F10 melanoma-bearing C57BL/6 mice. It could be effectively taken up by DCs and promote their maturation in lymph nodes. The nanovaccine increased neoantigen-specific T cells and upregulated the release of IFN-γ and TNF-α cytokines. The increased level of immune-suppressive molecules (Tregs and PD-L1) was detected after the immunization, and the vaccine-induced spleen lymphocytes have significant antitumor activity [134]. Meneveau et al. designed and evaluated an open-label, randomized phase I study of a transdermal vaccine comprising a mixture of twelve MHC class I-restricted melanoma peptides (originating from antigens of tyrosinase, gp100, MAGE-A1, MAGE-A3, MAGE-A10, and NY-ESO-1), a tetanus helper peptide (tet; AQYIKANSKFIGITEL), and GM-CSF. The study aimed to compare different adjuvants alone, or in combination, in order to decide which combination results in the best immunostimulatory effect. For this, DMSO and Montanide^TM^ ISA-51 alone or in combination with TLR 7 agonist imiquimod were used. It was found that CD8^+^ T cell responses were stronger in DMSO-containing groups alone or in combination with imiquimod (83% and 86% of participants, respectively), compared to Montanide^TM^ ISA-51 involved groups alone or in combination with imiquimod (28% and 14% of participants, respectively) [135].

#### 7.1.4. Poly-ICLC (aka Hiltonol^®^) 

Poly-ICLC is a double-stranded RNA (dsRNA) therapeutic compound with a combined immunostimulatory effect. It is composed of synthetic polyinosinic-polycytidylic acid (poly-IC) and two stabilizer compounds (to avoid enzymatic degradation, especially digestion by RNases), such as poly-L-lysine (PLL) and carboxymethylcellulose (CMC). Poly-IC is an immune stimulator with multiple effects; due to its pathogen-associated molecular pattern (PAMP), which mimics viral dsRNA properties, it can activate TLR 3 and cytoplasmic RNA helicases [136]. In Phase II, an open-label, randomized, two-arm study, the effects of different adjuvants were compared, where Arm A means Poly-ICLC-matured DC+PolyICLC and Arm B means Montanide ISA-51-VG+Poly-ICLC, as adjuvants for NY-ESO-1 and Melan-A/MART-1 long peptides in patients with melanoma in complete clinical remission but at high risk of disease recurrence (NCT02334735). It was found that the Arm B vaccine induced a stronger antibody and CD4^+^ T cell response, especially to NY-ESO-1, while the Arm A construct elicited CD8^+^ T cell responses against MelanA/MART1 compared to Arm A against both Melan-A/MART-1 and NY-ESO-1. The determination of the deeper molecular mechanism evoked by the vaccines, namely the correlation of HLA-type and cellular immune responses, is in progress [137].

#### 7.1.5. Immunostimulatory Proteins

Not only synthetic molecules, but often immunostimulatory proteins are also used in combination with antigens to boost the immune system. Since 1985, when granulocyte-macrophage colony-stimulating factor (GM-CSF) was identified, intensive studies have been driven to establish its mechanism of action. GM-CSF is frequently used for peptide vaccines due to its increased antibody production and CD8^+^ T cell-generating ability [120]. Based on the results obtained *in vivo* and during clinical trials, it has been established that the most promising results might be obtained by the application of different adjuvant combinations. One intensively developed group of adjuvants is using immunostimulating complexes (ISCOMs), which are cargo-like nanoparticles with approx. 40 nm size, and obtained a combination of antigens, cholesterol, phospholipid and the *Quillaja saponaria* saponin. This combination results in a hydrophobic interaction between the lipids and the antigen [138].

### 7.2. Vaccine Delivery Systems in Melanoma

In vaccines, the delivery system generally means different kinds of nanoparticles, which should protect the peptide vaccine from enzymatic degradation and also have depo-like effects; they ensure a continuous and sustained level of antigen at the injection site, thereby increasing the immune response. Due to their specific and different chemical and physiological properties, nanoparticles can influence their interaction with the target cells. The interaction can be easily optimized by the modification of the surface of nanoparticles (e.g., charge, structure, size, hydrophobicity, etc.) [139,140]; or by the conjugation of peptides (e.g., CPPS [141,142], APC-specific epitopes [143], or lipid moieties (e.g., lecithin)) [144]. In special cases, the delivery moiety has an adjuvant effect also; these are called vaccine-adjuvant delivery systems (VADS) [74,145]. In melanoma, five types of nanoparticles (liposomes, hydrogels, micelles, inorganic nanoparticles, and virus-like particles (VLPs)) have been reported as peptide vaccine delivery moieties [68], and among them, only two types have been tested in clinical trials (liposomes [146,147,148,149] and virus-like particles [150,151]. In addition, recent publications report an innovative new technique that could revolutionize immunotherapy and chemotherapy for melanoma. This technique is based on the use of microneedles, and their application for melanoma treatment was reviewed by Li and colleagues [152].

#### 7.2.1. Liposomes

Liposomes can be characterized by their lipid composition, surface charge, and size. All parameters can be easily changed by combining different lipids or by chemical modification to alter the surface charge (e.g., functionalization with carboxyl or amine groups) [153]. Chemical modifications not only affect the surface charge of liposomes but also allow conjugation to peptides, such as positively charged peptides or antigens [142,154,155,156]. It was found that positively charged liposomes may preferentially affect cellular uptake into APC-type macrophages and DCs and may induce a stronger immune response [120]. Lai et al. developed a DC-targeting vaccine against melanoma. In their construct, the liposome is coated with mannose, a well-known DC targeting molecule, and with the adjuvant CpG-ODN for delivering the melanoma antigen TRP-2(180–188) peptide. It was demonstrated that this vaccine composition significantly improved the antitumor immune response and increased the surviving of B16 melanoma cell-bearing mice. They suggested that the efficacy of the vaccine was mediated by the myeloid differentiation primary response gene 88 (MyD88) signaling pathway [157], which is an essential regulator of TLR-mediated signaling in DCs [158]. Due to their encapsulation and surface modification capabilities, they have also been successfully used as vaccine delivery systems in immunotherapy and combination therapy (chemo- and immunotherapy). Yazdani et al. reported that a cationic liposome decorated with gp100(25–33) antigen and combined with Cp, as an adjuvant, and with an anti-PD-1 monoclonal antibody, significantly promoted homing of DCs to tumor-draining lymph nodes, and increased the number of INF-γ producing CD8^+^ T cells in B16F10 melanoma tumor-bearing mice [159]. In another study, Su et al. designed cationic polymer-lipid hybrid nanovesicle-based liposomes as tumor vaccine delivery moiety. The vaccine consists of the octa-aspartic acid modified epitope (D_8_-conjugated SIINFEKL, which is an MHC I restricted melanoma antigen), CpG, and 1-methyl-tryptophan (1-MT), which is the inhibitor of the immune checkpoint indoleamine-2,3-dioxygenase (IDO). The construct can not only be effectively taken up by DCs and promote their maturation but also result in a CD8^+^ T cell response against B16-OVA tumor cells *in vitro.* Moreover, the vaccine significantly increased the recruitment of CD8^+^ and CD4^+^ T cells and inhibited tumor growth in tumor-bearing mice [160].

#### 7.2.2. Hydrogels 

Hydrogels are another type of delivery carriers that can be classified based on their origin: *natural* (including polysaccharides, polynucleotides, and polypeptides) or *synthetic* (consisting of synthetic polymers, e.g., poly-vinyl pyrrolidone (PVP), poly-lactic acid (PLA), poly-ethylene glycol (PEG), poly-vinyl alcohol (PVA), poly-acrylic acid (PAA), poly-acrylamide (PAAm), etc.), and *their combination* (e.g., collagen/matrigel, gelatin/alginate). Due to their highly porous construction, they form an extracellular matrix (ECM)-like structure that provides a stable repository for immunotherapy agents without damaging their biological activity [161]. The first immunotherapeutic application of hydrogels was in 2008, when DCs were injected into an injectable hydrogel matrix. The data demonstrate that hydrogels are not only efficient carriers but also capable of inducing a systemic immune response [162]. Hydrogels have been synthesized by different synthetic routes, and all of them have been carried out by crosslinking using γ-radiation, X-ray, hν, heating, or cross-linking agents [163,164]. The solubility of the hydrogels can be easily regulated by chemical modifications, especially by functionalization using polymers with different polarities or magnetic particles [165]. Due to their water-absorbing ability, hydrogels are well-dissolved in water and can enclose high-volume biological liquids and large amounts of molecules [166]. Nowadays, the release of drugs from hydrogels is precisely controlled due to the *in situ* gelation method, and many of them have injectable sol-gel properties [167,168].

Peptide-based hydrogels can be successfully used for cancer immunotherapy, they can deliver cytokines, anticancer vaccines, checkpoint inhibitors, and chimeric antigen receptor (CAR)-T cells [161]. Wakabayashi et al. developed a solid-in-oil nanodispersion carrier system for hydrophilic molecules. This nanostructure has high encapsulation ability and can effectively target APCs in the skin. They delivered a melanoma antigen (TRP-2(180–188)) with a solid-in-oil nanocarrier loaded with R-848 adjuvant (small TLR 7/8 agonist that induces cellular immunity) [169,170]. The obtained data suggested significant inhibition of melanoma growth and suppression of lung metastasis in tumor-bearing mice [171]. Dai et al. designed a series of hydrogel scaffolds that mainly consisted of melittin (26 amino acid long peptide from bee venom) and different lengths of RADA ([RADA]n, where n means the number of the tetrapeptide copies) self-assembling peptides. The MR_60_ (mellitin and RADA_8_) system has a direct antitumor effect and induces DC maturation. This construct was further developed, resulting in the MRK system, in which the hydrogel scaffold was composed of mellitin and RADA_6_. This MR_52_ hydrogel scaffold was loaded with a drug molecule (namely, KN93, which has a direct tumor-killing effect and induces the reprogramming of macrophages). It was found that MRK elicits a strong antitumor immune response and offers potent therapeutic efficacy for melanoma [172].

Since the development of the multiple antigen peptide system in 1988, which was a dendrimer structure from lysine monomers [173], a series of synthetic dendrimers can be found in the literature. The modification of MAPs with antigens (and/or adjuvants) was shown to impair immune-stimulatory properties essential for vaccine development [174]. The new generation of dendrimers is mainly composed of organic molecules with polymerization abilities. The synthetic, non-amino acid-based dendrimers are hyperbranched nanopolymers with a distinct, homogenous, and nearly monodisperse structure. They have a capacity for loading or conjugating of peptide antigens and immunostimulating molecules. They consist of a central core (which is an extended branch-like structure at repeating junctions) and reactive peripheral functional groups (that are suitable for conjugation). Dendrimers can be classified based on their synthetic route (convergent or divergent dendrimers) or based on their monomers. The most favorable dendrimer monomer units are polyamidoamine (PAMAM), arborols, polypropyleneimine (PPI), liquid crystalline, core–shell (tecto), and chiral dendrimers [120,175]. The main advantages of dendrimers are their high degree of branching, polyvalency, biocompatibility, and water solubility, which make them ideal carriers for various therapeutic agents. Despite their beneficial properties, only a few studies in the field of melanoma vaccines have demonstrated dendrimers as potential peptide antigen carriers. Xu et al. designed guanidinobenzoic acid modified polyamidoamine dendrimer (DGBA) as a carrier for peptide vaccines. They used OVA as the model antigen and CpG as an adjuvant to test the efficacy of their tumor vaccine construct in a B16-OVA melanoma model. They found that the vaccine induces a robust antigen-specific cellular immune response. Moreover, the vaccine had a significant therapeutic effect in combination with PD-1 immune checkpoint-blockade immunotherapy in the same *in vivo* model [176]. An excellent example of the combination of two kinds of delivery systems was introduced by Wang et al. They combined a four-arms PEG moiety with an injectable supramolecular hydrogel to form an effective delivery system for transferring adenovirus encoding Flagrp170 (a chimeric molecule which induces inflammatory cytokines, such as TNF-α and IFN-γ). The hydrogel-mediated release of the adenovirus vector resulted in an effective delivery of tumor antigens to target antigen-presenting cells and showed increased therapeutic efficacy in murine melanoma models [161,177].

#### 7.2.3. Micelles

Polymeric micelles are thermodynamically stable colloidal solutions that are generally formed by the self-assembly of amphiphilic block copolymers. Just as the other nanoparticle types, micelles can also enhance drug stability and efficacy by maintaining their delayed release. Micelles can be targeted to tumor cells; therefore, they can significantly reduce the toxicity of drugs [178]. Polymeric micelles as potential delivery units for metastatic melanoma treatment have huge importance. Zeng et al. introduced a melanoma antigen peptide, TRP-2 and CpG-ODN encapsulated hybrid polymeric micelle (formed by poly(ethylene glycol)phosphorethanolamine (PEG-PE) and a polyethylenimine-stearic acid conjugate), in order to address lymph nodes. The obtained results exactly demonstrated the improved targeting and anti-tumor effect of the hybrid polymeric micelle compared to the TRP-2 peptide alone, or in combination with CpG adjuvants without a delivery system [179]. In another study, TRP-2(180–188) (^180^SVYDFFVWL^188^) was complexed to the co-assembled nanoparticle, which was formed by conjugation of low-molecular-weight short-chain poly(ethylene glycol) (PEG, Mw 500) and pyropheophorbide-A (PPa) nanoconjugates, by hydrophobic interaction. This antigen-delivery nanoplatform is suitable for immunotherapy of melanoma through stimulation of DCs. It was found that the nanoplatform can itself, without any adjuvants, effectively be taken up by DCs and can cause their maturation *in vitro*. The nanoparticle pulsed DCs were injected into B16-F10 bearing C57BL/6 mice, resulting in a significant antitumor immune response with delayed tumor growth. Moreover, the nanoparticle has a self-chelating ability; therefore, the migration process and the biodistribution of the construct can be easily tracked by, e.g., PET imaging [180]. Chen and his co-workers demonstrated that non-antigenic epitopes can also elicit immune responses, depending on the delivery system. They reported a novel type of strategy for discovering self-assembly and self-adjuvant lipopeptides without saccharides as cancer vaccines. Their construct consists of a T cell helper peptide epitope and/or a hydrophobic fragment with the neoantigen epitope EGFRvIII. The non-antigenic EGFRvIII epitope was modified by lipids, such as cholesterol, which promotes a self-assembling property. The vaccine can trigger both humoral and cellular immune responses to EGFRvIII positive melanoma cells. It was also demonstrated that in *in vivo* melanoma models, self-adjuvant lipopeptide vaccine micelles efficiently prevented tumor growth, as well as tumorigenesis [100]. According to another study, micelles also have an effect on the remodeling of the tumor microenvironment. Huo et al. developed a multi-targeted receptor tyrosine kinase inhibitor (sunitinib base) encapsulated TRP-2 targeted polymeric micelle as a nano delivery system. Due to the targeting delivery of tyrosin kinase inhibitor by TRP-2 decorated polymeric micelle, a synergistic effect was observed which significantly enhanced the therapeutic efficacy of existing immune-based therapies for advanced melanoma [181]. Intensive research has started to develop new self-assembling nanostructures, both polymer-based and peptide-based ones. Although peptide nanocarriers might have huge relevance in tumor immunotherapy, some of them may trigger an undesired immune response. Therefore, there is a growing demand for the rational design of peptide-based nanocarriers with minimal inflammatory responses [182].

#### 7.2.4. Inorganic Nanoparticles

Due to their unique optical, physical, chemical, electronic, and magnetic properties, inorganic nanoparticles have been widely used for cancer diagnosis and treatment. Due to their biostability, biocompatibility, immunogenicity, and safety, some of them are used in preclinical experiments as vaccine delivery systems. The most commonly used inorganic nanoparticles against melanoma are gold and aluminum-based nanoparticles [68].

A DC-specific antibody-modified liposome-coated gold nanoparticle was introduced by Liang et al. for targeted delivery of MPLA adjuvant and TRP-2(180–188) melanoma antigen peptide. The construct, due to its efficient DC stimulatory and maturation ability, successfully activated CD8^+^ T cells, resulting in the inhibition of tumor growth, as well as metastasis in both B16-F10 prophylactic and lung metastasis models [183]. To avoid the unsuccessful clinical efficacy of peptide-based vaccines, Xu and co-workers designed and developed a therapeutic nanovaccine for melanoma immunotherapy by using gold-nanoparticle-conjugated peptides for incorporation with a supramolecular hydrogel for delivery of CpG adjuvant and antigen peptide (cysteine elongated chicken ovalbumin epitope (CSIINFEKL) was used as a model antigen) into a B16-OVA murine model. The injectable nanovaccine can target lymph nodes; there it activates and maturates APCs and induces a CD8^+^ T cell response with the association of a significantly increased number of tumor infiltrating CD8^+^ T cells. The vaccinated B16-OVA-bearing mice showed slower tumor progression with a prolonged survival rate [184]. Another example of a combination of two nanocarriers to increase the antigenicity of peptide antigens has been introduced by Zeng et al. They formed a host-guest self-assembly system between a non-covalently glycosylated gold nanoparticle and β-cyclodextrin (β-CD). The nanovaccine decorated with NY-ESO-1(157–162) melanoma antigen significantly suppressed tumor growth without any obvious toxicity, which suggested the improved therapeutic efficacy of antigens against melanoma in this newly developed nanoparticle [185].

As we discussed above, aluminum adjuvant has been used for more than 100 years and has an irreplaceable ability to stimulate the humoral immune response [186]. The idea of turning the classical gel-like Alum into a nano-sized vaccine carrier by spontaneous aggregation (aluminum-based nanoparticles, APNs) was reported by Jiang et al. They found that APNs can address the associated antigens in lymph nodes; they destabilize lysosomes, resulting in efficient cytosolic delivery and cross-penetration of antigens. The antigen and CpG-loaded APNs induce CD8^+^ T cell responses and prolong the survival of B16-OVA-bearing mice [187]. These impressive data have encouraged researchers to exploit the effectiveness of APNs and develop it further. Bai and colleagues used a PEG derivative stabilized APN for the delivery of a dual epitope peptide (ANLs), consisting of both MHC I and MHC II epitopes connected with an Arg_2_ spacer, in the presence of CpG ODN 1826 (*5′-tccatgacgttcctgacgtt-3′)*. The obtained results suggested that ANLs can elicit a double response, namely, they promoted the presentation of MHC I epitopes in APC and simultaneously activate and induce the proliferation of CD8^+^ T cells. Based on the *in vivo* data obtained after vaccination of B16-OVA and B16F10 mice, significantly decreased tumor growth and prolonged survival were observed in both models [188].

#### 7.2.5. Virus-like Particles 

**Figure 4:**
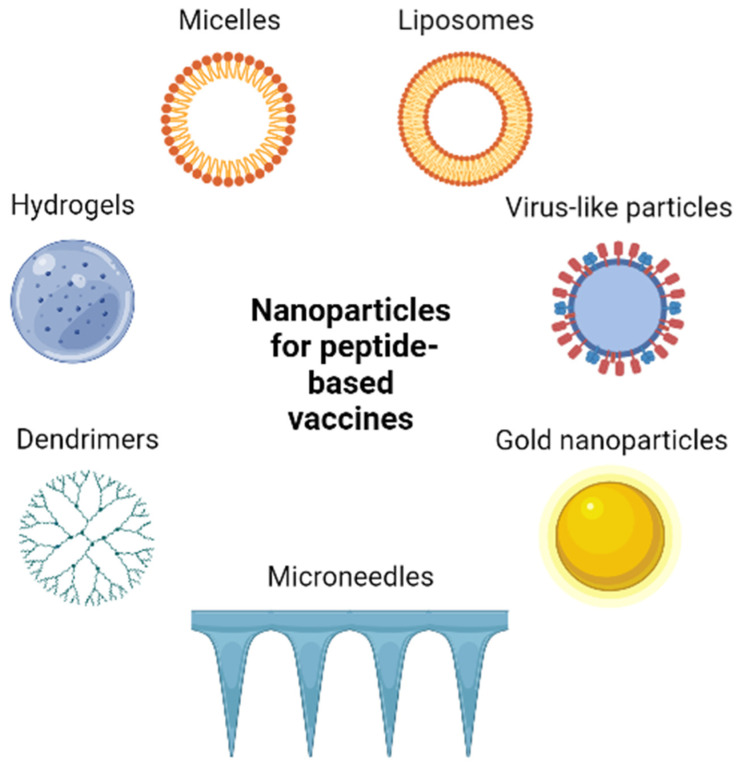
Nanoparticles for peptide-based vaccines in melanoma immunotherapy.

Viral capsid proteins are able to self-assemble into particle structures that closely resemble the natural virus from which they are derived. Virus-like particles (VLPs) are unable to replicate and are non-infectious, but they can efficiently deliver antigens to APCs, which are cross-presented in association with both MHC class I and class II, eliciting humoral and cellular immune responses, respectively [68]. Cheng and co-workers successfully prepared a hybrid VLP by reassembling of the Hepatitis B core protein (HBc). For the preparation, HBc monomers modified with two different tumor-specific antigens (HBc-gp100-6His monomers and HBc-OVA-6His monomers) were used. The hybrid VLPs elicit strong antitumor immune responses and significantly inhibit tumor growth and metastasis formation [189]. In a recent preclinical study, rabbit hemorrhagic disease virus (RHDV) VLP was decorated with gp100(25–33) melanoma-associated antigen immunodominant epitope to determine how functionalization can enhance the specific antitumor immune response. Therefore, they varied the number of copies of the epitope (one, two, or three copies), gp100(25–33) per VLP unit or functionalized the VLP surface to enhance the uptake by APCs. They found that although two or three copies of gp100(25–33) per VLP unit increased the IFN-γ production *in vitro*, this effect was diminished *in vivo*. The increasing number of epitopes per VLP subunit did not affect the activation of T cells *in vivo*. The functionalization of two copies of gp100(25–33) consisting of VPL surface by dimannosylation significantly enhanced the uptake by APCs due to the mannose-specific internalization way and resulted in delayed tumor growth and enhanced mouse survival [190]. In another interesting report, cucumber-mosaic virus-derived nanoparticles (CuMV-VLPs) were combined with the micro-size microcrystalline tyrosine (MCT) adjuvant to examine the effect of the particle size on the draining properties. The linkage between the VLP and epitope peptide (modified p33; H-**KAVYNFATM**GGCK(N_3_)-NH_2_, where the sequence corresponding to p33 is bold) was carried out by a Cu-free click reaction. The results obtained *in vivo* showed that formulating the CuMVTT-p33 nano-vaccine with MCT was more potent in blocking tumor growth compared to using the nanovaccine alone. The CuMVTT-p33 vaccine formulated with MCT adjuvant enhanced the level of infiltrating of CD8^+^ and p33-specific CTLs into the tumor, and the production of IFN-γ in B16F10p33 melanoma model [191]. An excellent example showed the relevance of VLPs in personalized immunotherapy against melanoma. Based on the ongoing clinical trials (NCT02680184, NCT03084640 and NCT03618641), in which a bacteriophage Qβ loaded with A-type CpGs nanoparticle have been tested with promising clinical prognosis. Mohsen and colleagues utilized a similar VLP which builds up a personalized multi-targeted vaccine based on identified and predicted mutated peptides coupled to TLR 9 ligand loaded Qβ VPL by Cu-free click chemistry. Three sets of multi-targeted vaccines were designed to immunize against the aggressive B16F10 murine melanoma: (1) germline epitope identified by immunopeptidomics, (2) mutated antigens predicted by whole exosome sequencing, and (3) a mixture of the identified germline and predicted mutated epitopes. It was found that all VLPs can elicit a CD8^+^ T cell response, but the best therapeutic effect was achieved with the mixed epitope, including VLP. Due to the Cu-free click chemistry, which is a non-toxic bio-orthogonal linkage method, the developed peptide-VLP platform has been ready for clinical translation of neoantigen-based personalized immunotherapy [192]. The main nanoparticles of peptide-based synthetic anti-cancer vaccines are shown in Figure 4.

## 8. Ongoing Clinical Trials

The high number of clinical trials related to peptide-based immunotherapies targeting melanoma also displays the relevance of peptide vaccines. Among the 3019 studies [193] that clinicaltrials.gov listed for melanoma-related studies, we found 593 hits for the search terms melanoma and immunotherapy [194], and 334 studies for the terms melanoma + vaccine [195]. When we added the search term ’peptide’ to the last two searches, we received 48 and 163 studies, respectively [196,197]. Among these 163 hits, 113 were completed [198] (21 studies with results [199]); 121 studies included patients with Stage IV melanoma [200], and 116 studies targeted metastatic melanoma [201]. Most of them were Phase 1 and Phase 2 studies (91 and 94 studies, respectively) [202,203]. In Table 2, we summarized the ongoing clinical trials that have already been completed, but the data analysis and follow-up studies are continuously evaluated and published on the clinicaltrials.gov website (accessed on 11 December 2022). The table includes studies that were last updated in 2017 or later. 

Data from clinical trials show that only small successes are achieved with peptide-based vaccines, but a breakthrough is still a long way off. Several processes may play a role, either together or separately, in the background of this failure. In most of the clinical trials, multiepitope vaccines have been used for the treatment of melanoma, which can reduce the risk of immune escape [216]. Unfortunately, peptide-based vaccines can eliminate only a small number of tumor cells due to the continuously altered antigen set of tumors. Regrettably, not only the tumor antigen-based vaccines but also the neoantigen-containing ones were ineffective for complete tumor elimination. Many types of mutations are generated, cloned, altered, and lost from the tumor cell genome during tumorigenesis. Due to the tumor heterogenicity, these mutations are also different in the individual tumor cells, resulting in different antigens and neoantigens. On the other hand, the neoantigens, in most cases, have insufficient maturity, which might be the main reason for their ineffectiveness [217]. 

The immune escape of tumor cells is the other critical issue hindering the efficacy of peptide-based vaccines. Tran and coworkers established that loss of HLA site heterozygosity strongly limits clinical responses to either peptide-based vaccines or adoptive T cell therapies [218]. In addition to antigen loss, tumor cells have various complex immune escape mechanisms, including suppression of immune checkpoints (PD-1, CTLA-4), immunosuppression of various cells in the tumor microenvironment [120], and release of ions or proteins inside tumor cells after necrosis, all of which compromise the recognition and activation of neoantigens by T cells. Peptide-based vaccines, in combination with other conventional therapies, such as chemoradiotherapy and targeted therapy, show great potential for development [217].

Another possible reason for ineffective melanoma treatment is the phenomenon of epitope spreading. This process can be induced by either vaccination, applying immune checkpoint inhibitors, and/or adoptive T cell therapy [219]. In all cases, treatment results in the lysis of tumor cells. The dying tumor cells or their components are taken up by APCs, which generate new antigens and present them to T cells, which migrate to the tumor and destroy it. These newly generated antigens result in T cell proliferation and the release of a cascade of cytokines and chemokines that participate in the differentiation, activation, and recruitment of additional T and B cell populations, as well as myeloid cells and APCs, which determine the immune response. This novel antigen presentation pathway has been termed as cross-presentation.

Last, but not least, the following parameters must be kept in mind when trying to find a way to maximize the effectiveness of the vaccination: (i) What is the general state of health of the patient? (ii) Which tumor stage (primary or metastatic) can be determined? (iii) Can we identify prognostic factors or predictive biomarkers? [216].

Considering the above-described reasons, the combination of tumor vaccines with other traditional therapies, such as chemoradiotherapy and targeted therapy, might be the solution to the ineffectiveness of melanoma treatment.

## 9. Conclusions

Cancer vaccines, especially peptide-based vaccines, have emerged and developed rapidly in the last few decades. They are based on tumor antigens that can activate and maturate DCs; therefore, targeting DCs is one of the most important goals for vaccine development, which can be achieved by several kinds of chemical alterations of possible antigens, such as modification of their structure, using conjugation strategies, or encapsulation into nanoparticles. Peptide-based vaccines are produced almost exclusively by synthetic methods and can be fully and accurately characterized as chemical entities. Due to synthetic approaches, the production of peptide vaccines is simple, easily variable, and reproducible. Despite the synthetic advantages, peptide antigens are poorly immunogenic themselves; the use of adjuvants is essential for eliciting a high-frequency immune response. Unfortunately, adjuvants alone cannot increase the immunogenicity of peptide antigens; the use of delivery systems is usually required to achieve the desired response. The diverse possibilities to chemically modify the peptide structure; the versatile conjugation methods, along with the numerous possibilities of adjuvant usage and delivery options, make the topic of anti-cancer peptide vaccines a dynamic and constantly developing field. Moreover, the continuously increasing number of clinical trials related to peptide-based immunotherapies targeting melanoma unequivocally displays the relevance of peptide vaccines.

## Figures and Tables

**Table 1:** Advantages and drawbacks of peptide-based anti-tumor vaccines.

Advantages	Drawbacks
Simple, reproducible, cost-effective production	Poorly immunogenic
Chemically and physically fully and thoroughly characterizable	Require the use of adjuvants and delivery systems
Non-natural amino acids and peptidomimetics can be introduced	Susceptibility for enzymatic degradation
Lipids, carbohydrate and phosphate groups can be incorporated	Unrelated immune responses during degradation
Does not require expression: pure and easily detectable product	Often not uniformly recognized by all patients
Large-scale production can be economically carried out	
Usage of nanoparticles: water solubility and stability can be reached	
Safe storage after lyophilization	
Biodegradability	
Possibility of multiepitope peptides	

**Table 2:** Ongoing clinical trials of peptide-based vaccines.

NCT Number	Stage of Melanoma	Vaccine Composition	Groups	Completed Clinical Trial	Ref
NCT00254397	not labelled	drug: Leuprolide (D) antigen(s): gp100(209–217) (Gp209), MAGE-3 peptide (M3) adjuvant(s): -	Group 1: D+Gp209 Group 2: Gp209 Group 3: Gp209+M3+D Group 4: Gp209+M3	Phase II	[204]
NCT00118274	II III IV	drug: Cyclophosphamide (D) antigen(s): melanoma helper peptide (MHP), multiepitope melanoma peptide (MeMP), vaccine tetanus toxoid helper peptide (TTHP) adjuvant(s): incomplete Freund’s adjuvant (IFA), Montanide ISA-51 (ISA)	Group 1: MeMP+TTHP+IFA Group 2: D+MeMP+TTHP+IFA Group 3: MHP+MeMP+ISA Group 4: D+MHP+MeMP+ISA	Phase I Phase II	[205]
NCT01876212	not labelled	drug: Dasatinib (D) antigen(s): αDC1 ^1^ incorporation with TBVA ^2^-derived peptides (DC+TBVA) adjuvant(s): -	Group 1: D+(DC+TBVA) (cycle 1+cycle2) Group 2: D+(DC+TBVA) (cycle 1+cycle1)	Phase II	[206]
NCT00003895	IA, IB, IIA, IIB, IIC, IIIA, IIIB, IIIC	drug: - antigen(s): HPV16E7(12–20) ^3^ (HPV12), gp100(209–217) (Gp209) adjuvant(s): Montanide ISA-51 (ISA)	Group 1: p209+HPV12+ISA in every 2 weeks for 6 months Group 2: Gp209+HPV12+ISA in every 3 weeks for 6 months	Phase II	[207]
NCT00960752	not labelled	drug: - antigen(s): gp100(209–217) (Gp209), MAGE-3 (M3) adjuvant(s): Resiquimod (R848)	Group 1: Gp209+M3+R848 Group 2: Gp209+M3 Group 3 (Metastatic melanoma patients): Gp209+M3+R848	Phase II	[208]
NCT01961115	IIIA, IIIB, IIIC IV	drug: Epacadostat (D) antigen(s): multiepitope peptide antigens (MELITAC 12.1) adjuvant(s): -	Group 1: D+MELITAC 12.1	Phase II	[209]
NCT00084656	III IV	drug: ipilimumab (D) antigen(s): multiepitope melanoma peptide(MeMP) ^4^ adjuvant(s): Montanide ISA-51 (ISA)	Group 1: D+MeMP+ISA	Phase II	[210]
NCT01989572	IIA, IIB, IIC, IIIA, IIIB, IIIC, IV	drug: - antigen(s): multiepitope melanoma peptide(MeMP) ^4^ adjuvant(s): GM-CSF, Montanide ISA-51 (ISA)	Group 1: MeMP+GM-CSF+ISA Group 2: MeMP+placebo Group 3: GM-CSF+placebo Group 4: placebo+placebo	Phase III	[211]
NCT00019682	IIIA, IIIB, IIIC, IV	drug: - antigen(s): gp100(209–217) (Gp209) Cytokine: IL2 adjuvant(s): Montanide ISA-51 (ISA)	Group 1: IL2 Group 2: Gp209+ISA+IL2	Phase III	[212]
NCT00112242	not labelled	drug: - antigen(s): Melan-A analogs (ELA or EAA), NY-ESO-1 analogs (b and LP), MAGE-A10, gp100(209–217) (Gp209) Cytokine: IL2 adjuvant(s): Montanide ISA-51 (ISA), CpG	Group 1: ELA + ISA Group 2: ELA+ b+MAGE-A10+ISA Group 3: EAA+ELA+ Mage-A10+b+ISA+CpG Group 4: ELA+Mage-A10+LP+ISA+CpGGroup 5: EAA+ELA+ Mage-A10+LP+ISA+CpG+IL2		[213]
NCT00518206	not labelled	drug: Cyclophosphamide (D) antigen(s): NY-ESO-1 analogs Cytokine: IL2 adjuvant(s): ISOMATRIX^®^	Group 1: NY-ESO-1+ISOMATRIX^®^ Group 2: D+ NY-ESO-1+ISOMATRIX^®^	Phase II	[214]
NCT00142454	not labelled	drug: Imiquimod (D) antigen(s): NY-ESO-1 analogs adjuvant(s): -	Group 1: D+NY-ESO-1	Phase I	[215]

^1^ autologous type-1 polarized Dendritic Cell; ^2^ tumor blood vessel antigen; ^3^ human papillomavirus (HPV) 16 E7(12–20) peptide; ^4^ consisting of TYR, gp100 and MART-1 antigens.
